# Variation in diurnal sedation in mechanically ventilated patients who are managed with a sedation protocol alone or a sedation protocol and daily interruption

**DOI:** 10.1186/s13054-016-1405-3

**Published:** 2016-08-01

**Authors:** Sangeeta Mehta, Maureen Meade, Lisa Burry, Ranjeeta Mallick, Christina Katsios, Dean Fergusson, Peter Dodek, Karen Burns, Margaret Herridge, John W. Devlin, Maged Tanios, Robert Fowler, Michael Jacka, Yoanna Skrobik, Kendiss Olafson, Deborah Cook, for the SLEAP Investigators and the Canadian Critical Care Trials Group

**Affiliations:** 1 Department of Medicine and Interdepartmental Division of Critical Care Medicine, Mount Sinai Hospital and University of Toronto, Toronto, Ontario Canada; 2 Departments of Medicine, Clinical Epidemiology and Biostatistics, McMaster University, Hamilton, Ontario Canada; 3 Department of Pharmacy and Medicine, Mount Sinai Hospital and University of Toronto, Toronto, Ontario Canada; 4 Clinical Epidemiology Program, Ottawa Hospital Research Institute and Faculty of Medicine, University of Ottawa, Ottawa, Ontario Canada; 5 Interdepartmental Division of Critical Care Medicine, University of Toronto, Toronto, Ontario Canada; 6 Division of Critical Care Medicine and Center for Health Evaluation and Outcome Sciences, St. Paul’s Hospital and University of British Columbia, Vancouver, British Columbia Canada; 7 Department of Critical Care, St. Michael’s Hospital, Toronto, Ontario Canada; 8 Li Ka Shing Institute, Toronto, Ontario Canada; 9 Department of Medicine and Interdepartmental Division of Critical Care Medicine, University Health Network and University of Toronto, Toronto, Ontario Canada; 10 School of Pharmacy, Northeastern University, Boston, Massachusetts USA; 11 Department of Medicine, Long Beach Memorial Medical Center, Long Beach, California USA; 12 Departments of Medicine and Critical Care Medicine, Sunnybrook Health Sciences Centre, Toronto, Ontario Canada; 13 Department of Anesthesiology, University of Alberta Hospitals, Edmonton, Alberta Canada; 14 Department of Medicine, Hôpital Royal Victoria, Montréal, Quebec Canada; 15 Section of Critical Care, Department of Medicine, University of Manitoba, Winnipeg, Manitoba Canada; 16 Departments of Medicine, Clinical Epidemiology & Biostatistics, McMaster University, St Joseph’s Healthcare, Hamilton, Ontario Canada; 17 Mount Sinai Hospital, Suite 18-216, 600 University Ave., Toronto, M5G 1X5 Canada

**Keywords:** Sedation, Opioids, Mechanical ventilation, Protocols, Weaning, Diurnal rhythm, Intensive care unit

## Abstract

**Background:**

Mechanically ventilated patients may receive more sedation during the night than during the day, potentially delaying extubation. We compared nighttime and daytime benzodiazepine and opioid administration in adult patients enrolled in a multicenter sedation trial comparing protocolized sedation alone or protocolized sedation combined with daily sedation interruption; and we evaluated whether nighttime and daytime doses were associated with liberation from mechanical ventilation.

**Methods:**

This is a secondary analysis of a randomized trial which was conducted in 16 North American medical-surgical ICUs. In all 423 patients, nurses applied a validated sedation scale hourly to titrate benzodiazepine and opioid infusions to achieve a light level of sedation. Using fentanyl equivalents and midazolam equivalents, we compared dosages administered during night (19:00 to 07:00) and day (07:00 to 19:00) shifts. Using multivariable logistic regression we evaluated the association between nighttime and daytime opioid and sedative doses, and spontaneous breathing trial (SBT) conduct, SBT success, and extubation.

**Results:**

Nighttime benzodiazepine and opioid doses were significantly higher than daytime doses (mean difference midazolam equivalents 23.3 mg, 95 % CI 12.9, 33.8, *p* < 0.0001; mean difference fentanyl equivalents 356 mcg, 95 % CI 130, 582, *p* = 0.0021). Mean Sedation Agitation Scale score was similar between night and day, and was at target (3.2 vs 3.3, 95 % CI −0.05, 0.02, *p* = 0.35). Self-reported nurse workload was similar during the night and day. Patients were more often restrained during day shifts (76.3 % vs 73.7 %, *p* < 0.0001), and there were more unintentional device removals during the day compared with night (15.9 % vs 9.1 %, *p* < 0.0001). Increases in nighttime drug doses were independently associated with failure to meet SBT screening criteria, SBT failure, and the decision not to extubate the patient despite successful SBT.

**Conclusion:**

Patients received higher doses of opioids and benzodiazepines at night. Higher nighttime doses were associated with SBT failure and delayed extubation.

**Trial registration:**

ClinicalTrials.gov NCT00675363. Registered 7 May 2008.

**Electronic supplementary material:**

The online version of this article (doi:10.1186/s13054-016-1405-3) contains supplementary material, which is available to authorized users.

## Background

Analgesia and sedation are a ubiquitous and essential component of care for critically ill patients. However, deep sedation is associated with prolonged mechanical ventilation (MV), longer intensive care unit (ICU) stay [1, 2], and higher mortality [3, 4]. Mechanically ventilated patients may receive more sedation during the night compared with the day [5], potentially delaying extubation [6]. Diurnal variation in sedation in critically ill patients is important to elucidate as it may affect weaning from mechanical ventilation, impair cognition, and worsen sleep [7].

SLEAP was a multicenter trial that randomized mechanically ventilated adult patients to protocolized sedation (PS) alone (control), or protocolized sedation combined with daily interruption (DI) of sedation [8]. There was no difference between the two groups in the primary outcome of time to extubation (hazard ratio (HR) 1.08, 95 % CI 0.86, 1.35, *p* = 0.52), nor in secondary outcomes of ICU and hospital length of stay [9]. However, the DI group received higher doses of opioids and benzodiazepines, potentially reflecting nurse apprehension about patient discomfort or the risk of adverse events during DI.

The objective of this study was to describe daytime and nighttime doses of sedatives and opioids, and to identify associations between these doses and conduct of spontaneous breathing trials (SBTs), success of SBTs, and extubation, in patients enrolled in the SLEAP Trial. We hypothesized that patients received more opioids and sedatives at night than during the day, regardless of randomization group, and that higher nighttime doses would be associated with delays in the extubation process.

## Methods

This was a secondary analysis of the multicenter randomized SLEAP trial [8]. We included critically ill adults who were expected to require mechanical ventilation for longer than 48 hours and were receiving continuous infusions of opioids and/or benzodiazepines. Patients admitted to the ICU after cardiac arrest or traumatic brain injury, patients receiving neuromuscular blockade, or patients without a commitment to maximal therapy were excluded. The Research Ethics board at each participating site approved the study, and written informed consent was obtained from substitute decision makers.

In both study arms the Sedation Agitation Scale (SAS) or Richmond Agitation Sedation Scale (RASS) were recorded hourly, and nurses titrated opioid and sedative infusions to achieve a target SAS score of 3 to 4, or RASS score of −3 to 0. In the DI arm nurses interrupted benzodiazepine and opioid infusions daily; if ongoing infusions were necessary they were resumed at half the previous dose(s). If infusions were no longer necessary patients were managed with intermittent doses of opioids and sedatives. Mechanical ventilation was weaned at the discretion of the ICU team. As previously described [2], patients in both arms were screened daily by respiratory therapists for eligibility to have an SBT; information about successful SBTs was communicated to the ICU team with a view to extubation. Decisions on extubation were at the discretion of the ICU team.

We recorded total doses of sedatives and opioids administered to patients during mechanical ventilation, as infusions and intermittent bolus doses. We calculated total medication doses and number of bolus doses administered during night shifts and day shifts. Night shifts were defined as 12 hours from 19:00 to 07:00 hours, and day shifts were defined as 12 hours from 07:00 to 19:00 hours.

Twice daily at the end of each shift, bedside nurses recorded their perceived additional clinical workload related to trial procedures, using a 10-point visual analog scale (VAS), with 1 corresponding to “very easy” and 10 corresponding to “difficult”. Daily, we recorded physical restraint use and unintentional device removal during each shift.

### Statistical analysis

Descriptive data are presented as percentages for categorical data, means with standard deviations for normally distributed variables, and medians with interquartile ranges for non-normally distributed variables. For the analysis, opioid doses were converted to fentanyl equivalents (10 mg morphine = 2 mg hydromorphone = 0.1 mg fentanyl) and benzodiazepine doses were converted to midazolam equivalents (1 mg midazolam = 0.5 mg lorazepam) [8]. RASS scores were converted to an SAS equivalent [8] (Additional file 1, Table 1).

**Table 1:** Baseline characteristics of patients

Characteristic	Protocolized sedation and daily interruption	Protocolized sedation
	*n* = 214	*n* = 209
Age, years, median (IQR)	57 (46–70)	60 (49–70)
Female sex, *n* (%)	93 (44)	92 (44)
Type of admission, *n* (%)		
Medical	175 (82)	179 (86)
Surgical	30 (14)	22 (11)
Trauma	8 (4)	6 (3)
Body mass index, median (IQR)	28.2 (23.8–34.2)	28.6 (25.0–33.2)
APACHE II, median (IQR)	24 (18–28)	23 (19–29)
Mechanical ventilation days, median (IQR)	2 (1–4)	2 (1–4)
Pre ICU conditions, *n* (%)		
Alcohol use	49 (23.0)	44 (21.2)
Tobacco use	48 (22.5)	40 (19.3)
Any psychiatric condition	42 (19.6)	29 (14.4)
Any neurologic condition	33 (15.4)	36 (17.2)
Respiratory disease	17 (8.0)	26 (12.4)
Renal dysfunction	20 (9.4)	16 (7.7)
Habitual drug use	14 (6.6)	10 (4.8)
Liver disease	12 (5.6)	11 (5.3)

Baseline demographic data for patients randomized to each arm of the SLEAP study. There were no significant differences between the two groups. Pre ICU conditions: neurological condition defined as stroke, seizure disorder, dementia, neuromuscular disease, Parkinson’s disease, or other neurological condition; psychiatric condition includes depression, bipolar disorder, schizophrenia, anxiety disorder, or other psychiatric condition; respiratory disease defined as home oxygen, CO_2_ retention at baseline, or home ventilation; renal dysfunction defined as chronic renal failure with creatinine >180 umol/L, or chronic dialysis; habitual drug use other than tobacco or alcohol; liver disease defined as Child Pugh grade C or known esophageal varices. *IQR* interquartile range, *APACHE II* Acute Physiology and Chronic Health Evaluation II.

Using multivariable logistic regression with generalized estimating equations (GEE) to account for repeated measurements within patients, we evaluated the association between the nighttime and daytime opioid and sedative doses on the previous study day with three different outcomes: 1) meeting criteria to have an SBT; 2) passing the SBT; and 3) not being extubated despite passing the SBT. The baseline covariates included in our model were randomization group, age, sex, and medical vs surgical diagnosis. In order to account for the time-dependent nature of sedative exposure, our model accounted for the daytime benzodiazepine dose on the previous study day, the difference between nighttime and daytime benzodiazepine dose, daytime opioid dose, and difference between nighttime and daytime opioid dose, for every patient. All statistical tests were two sided, with a *p* value <0.05 considered statistically significant. All statistical analysis was done using SAS 9.3 (SAS Institute Inc., Cary, NC, USA).

## Results

Among 423 patients enrolled in the SLEAP study, patient characteristics were similar between the DI and control groups. The mean APACHE II scores were 24 and 23, respectively (Table 1), 84 % had medical diagnoses and, at enrollment, patients had been mechanically ventilated for a median of 2 days.

### Nighttime and daytime benzodiazepine and opioid dosing

**Fig. 1:**
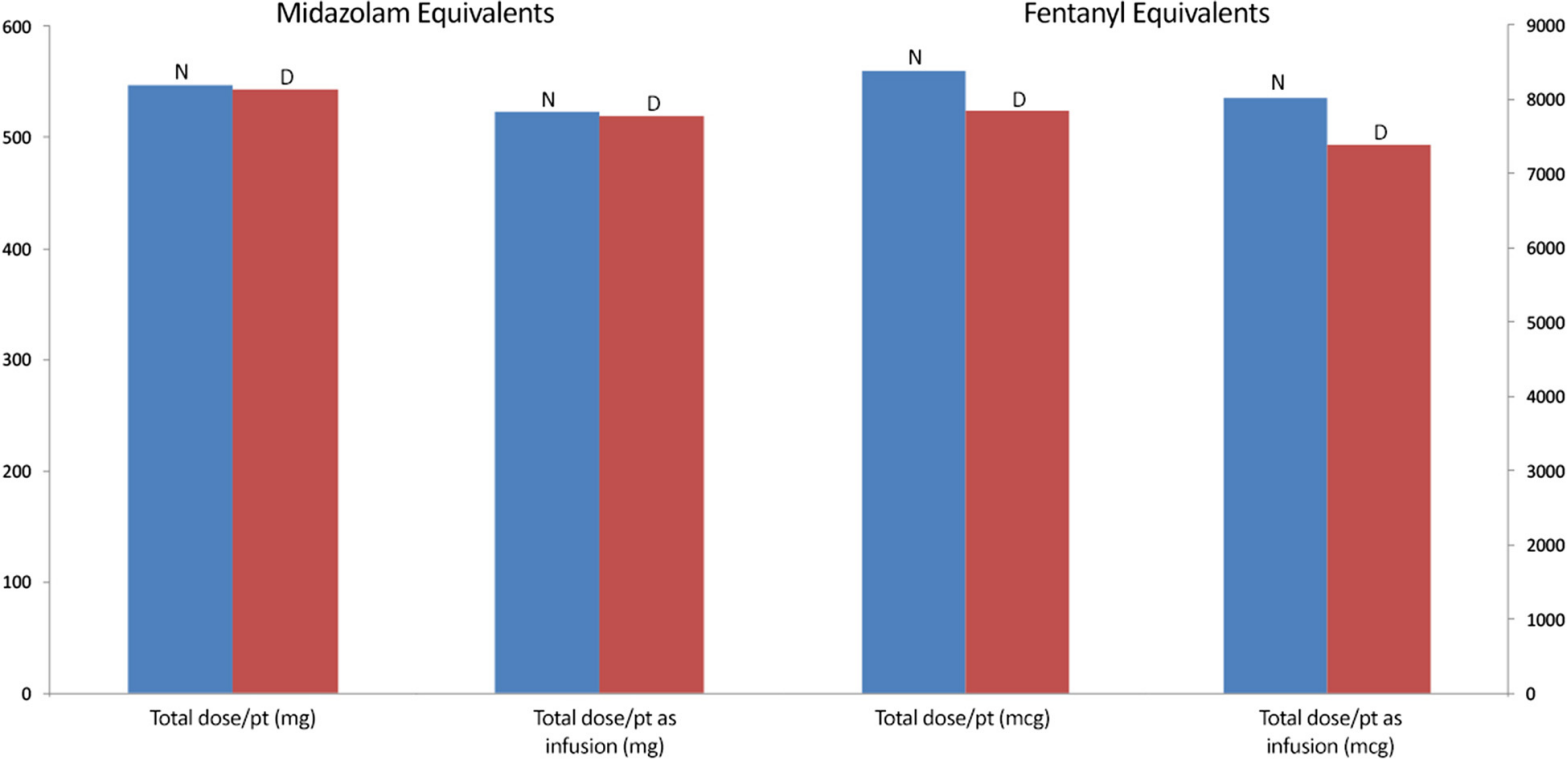
Daily benzodiazepine and opioid doses during night and day shifts. Mean benzodiazepine (midazolam equivalents, mg) and mean opioid (fentanyl equivalents, mcg) administration for all patients during night (*N*, *blue bars*, 19:00–07:00 hours) and day (*D*, *red bars*, 07:00–19:00 hours) shifts. *Total dose/patient* represents doses received for the duration of the study; *Total dose/patient as infusion* represents total doses received through intravenous infusion, excluding bolus doses, for the duration of the study. *P* < 0.005 for all four comparisons of nighttime versus daytime doses

Among all 423 patients, nighttime benzodiazepine and opioid doses were significantly higher than daytime doses (mean difference midazolam equivalents 23.3 mg, 95 % confidence interval (CI) 12.9, 33.8, *p* < 0.0001; mean difference fentanyl equivalents 356 mcg, 95 % CI 130, 582, *p* = 0.0021) (Fig. 1 and Table 2). Although patients received more opioid bolus doses during the day than during the night, there was no difference in the number of benzodiazepine bolus doses administered during night and day shifts.

**Table 2:** Nighttime vs daytime benzodiazepine and opioid administration in all 423 patients

	Nighttime	Daytime	Mean Difference (95 % CI)	*P* value
Midazolam equivalents				
Total dose/pt (mg)^a^	547 (2220)	523 (2236)	23.3 (12.9, 33.8)	<0.0001
Total dose/pt, infusion (mg)	543 (2216)	519 (2231)	23.8 (13.1, 34.5)	<0.0001
Total dose/pt, bolus (mg)	4.0 (16.4)	4.5 (16.4)	−0.5 (−1.5, 0.6)	0.37
Number of boluses^b^	1.2 (4.0)	1.2 (3.9)	0.02 (−0.2, 0.2)	0.81
Bolus dose (mg)	0.39 (1.4)	0.43 (1.5)	−0.04 (−0.1, 0.1)	0.41
Fentanyl equivalents				
Total dose/pt (mcg)^a^	8379 (22754)	8024 (23083)	356 (130, 582)	0.002
Total dose/pt, infusion (mcg)	7846 (22270)	7387 (22539)	459 (236, 682)	<0.0001
Total dose/pt, bolus (mcg)	534 (914)	637 (1082)	−103 (−150, −55)	<0.0001
Number of boluses^b^	11.0 (14.8)	12.1 (15.9)	−1.2 (−1.8, −0.5)	0.0001
Bolus dose (mcg)	51.4 (71.6)	52.4 (59.9)	−1.0 (−6.7, 4.7)	0.73

Benzodiazepine (midazolam equivalents) and opioid (fentanyl equivalents) administration for all patients during night (19:00–07:00 hours) and day (07:00–19:00 hours) shifts. All data are presented as mean (SD). Mean difference is presented for total dose/patient (Total dose/pt), and all routes of administration including boluses and infusions. ^a^Total dose received over the duration of the study. ^b^Total number of boluses received over the duration of the study

### Nighttime and daytime dosing in the daily interruption versus control groups

We compared benzodiazepine and opioid doses administered to the DI and control groups (Additional file 1, Table 2). Compared with the control group, the DI group received higher benzodiazepine doses during both day (mean difference 9 mg, 95 % CI 0.1, 18.0, *p* = 0.047) and night (mean difference 12 mg, 95 % CI 2.7, 21.1, *p* = 0.01) shifts, and higher opioid doses during both day (mean difference 336 mcg, 95 % CI 236, 436, *p* < 0.0001) and night (mean difference 405 mcg, 95% CI −48, 1636, *p* < 0.0001) shifts. In terms of bolus dosing, the DI group also received more opioid boluses during both day and night shifts, and more benzodiazepine boluses at night.

We further compared the total doses (infusion and bolus) administered during the night and day within each randomization group (Additional file 1, Table 3). In the DI group, mean total opioid and benzodiazepine doses administered per shift were higher at night than during the day. The control group also received more benzodiazepines and opioids at night than during the day. Patients in the DI group received more opioid boluses during the daytime compared with nighttime.

**Table 3:** SAS scores, VAS scores and unintentional device removal during night and day shifts

	Nighttime	Daytime	*P* value
SAS score, mean (SD)	3.2 (0.8)	3.3 (0.8)	0.35
SAS score, number of values	49437	49264	
Nurse VAS score, mean (SD)	3.9 (1.4)	4.0 (1.4)	0.38
Nurse VAS score, number of values	4179	4471	
Patients with physical restraint, *n* (%)	308 (73.7)	321 (76.3)	<0.0001
Shifts with physical restraint, median (IQR)	3 (0,6)	3 (1,7)	<0.0001
Unintentional device removal, *n* (%)			
At least one	38 (9.1)	67 (15.9)	<0.0001
Central venous catheter	2 (0.5)	5 (1.2)	<0.0001
Arterial catheter	10 (2.4)	14 (3.3)	<0.0001
Endotracheal tube	7 (1.7)	17 (4.0)	<0.0001
Gastric tube	24 (5.7)	35 (8.3)	<0.0001
Urinary catheter	6 (1.4)	13 (3.1)	<0.0001
Peripheral venous catheter	4 (1.0)	9 (2.1)	<0.0001

The Riker Sedation Agitation Scale (SAS) score and nurse visual analog scale (VAS) score represent workload associated with trial procedures, use of physical restraint, and unintentional device removal during night and day shifts. *IQR* interquartile range

### SAS scores, nurse workload, device removal, and use of physical restraint

Mean reported SAS scores did not differ between night and day shifts, and were within the target range (3.2 vs 3.3, respectively, mean difference −0.02, 95 % CI −0.05, 0.02, *p* = 0.35) (Table 3). Self-reported nurse workload using a VAS (score 1–10) was similar during the night and day (3.9 vs 4.0, mean difference −0.04, 95 % CI −0.05, 0.12, *p* = 0.38). There were more unintentional device removals (self-extubation, removal of venous access) during the day compared with the night (15.9 % vs 9.1 %, *p* < 0.0001). The majority of patients (78 %) were restrained at least once during the study, more often during day shifts than during night shifts.

### Multivariable analyses

Factors independently associated with a patient meeting the criteria for an SBT (Table 4) were: lower daytime midazolam dose (odds ratio (OR) 0.9930, 95 % CI 0.9892, 0.9969, *p* = 0.0004), smaller difference between nighttime and daytime midazolam dose (OR 0.9938, 95 % CI 0.9896, 0.9980, *p* = 0.0004), and lower daytime fentanyl dose (OR 0.9402, 95 % CI 0.9071, 0.9746, *p* = 0.0008).

**Table 4:** Variables associated with the patient meeting criteria for a spontaneous breathing trial, multivariable analysis

	Odds ratio	95 % CI		*P* value
		lower	upper	
Sedation protocol and daily interruption vs sedation protocol alone	0.956	0.685	1.332	0.79
Age (10-year increase)	1.013	0.892	1.151	0.84
Gender (male vs female)	1.039	0.734	1.474	0.83
Surgical/trauma vs medical	0.799	0.441	1.446	0.46
Daytime midazolam dose (1 mg increase)	0.993	0.989	0.997	0.0004
Midazolam: nighttime-daytime dose (1 mg increase)	0.994	0.989	0.998	0.0004
Daytime fentanyl dose (100 mcg increase)	0.940	0.907	0.975	0.0008
Fentanyl: nighttime-daytime dose (100 mcg increase)	0.977	0.938	1.018	0.27

Passing an SBT (Table 5) was independently associated with lower daytime fentanyl dose (OR 0.9602, 95 % CI 0.9347, 0.9864, *p* = 0.003), and a smaller difference between nighttime and daytime fentanyl dose (OR 0.9729, 95 % CI 0.9524, 0.9937, *p* = 0.01).

**Table 5:** Variables associated with the patient passing the spontaneous breathing trial, multivariable analysis

	Odds ratio	95 % CI		*P* value
		lower	upper	
Sedation protocol and daily interruption vs sedation protocol alone	1.206	0.828	1.756	0.33
Age (10-year increase)	0.949	0.839	1.075	0.42
Gender (male vs female)	1.030	0.716	1.483	0.87
Surgical/trauma vs medical	1.598	0.884	2.891	0.12
Daytime midazolam dose (1 mg increase)	0.998	0.994	1.001	0.13
Midazolam: nighttime-daytime dose (1 mg increase)	0.997	0.992	1.002	0.28
Daytime fentanyl dose (100 mcg increase)	0.960	0.935	0.986	0.003
Fentanyl: nighttime-daytime dose (100 mcg increase)	0.973	0.952	0.994	0.01

The only factor independently associated with a patient not being extubated after passing an SBT (Table 6) was a larger difference between nighttime and daytime midazolam dose (OR 1.0145, 95 % CI 1.0039, 1.0253, *p* = 0.007), with more midazolam given at night than during the day.

**Table 6:** Variables associated with the patient not being liberated from mechanical ventilation despite passing the spontaneous breathing trial, multivariable analysis

	Odds ratio	95 % CI		*P* value
		lower	upper	
Sedation protocol and daily interruption vs. sedation protocol alone	0.957	0.644	1.421	0.83
Age (10-year increase)	1.124	0.987	1.280	0.08
Gender (male vs female)	1.420	0.967	2.086	0.07
Surgical/trauma vs medical	1.066	0.630	1.804	0.81
Daytime midazolam dose (1 mg increase)	1.008	0.999	1.017	0.07
Midazolam: nighttime-daytime dose (1 mg increase)	1.014	1.004	1.025	0.007
Daytime fentanyl dose (100 mcg increase)	1.012	0.961	1.067	0.64
Fentanyl: nighttime-daytime dose (100 mcg increase)	0.974	0.906	1.047	0.48

## Discussion

In the SLEAP study, mechanically ventilated adult patients in both arms of the trial received more opioids and benzodiazepines during the night compared with during the day. Increased nocturnal sedation was independently associated with failure to meet criteria for an SBT, failure to pass the SBT, and delayed extubation after passing an SBT.

Greater daytime patient wakefulness is also suggested by more unintentional device removals (self-extubation, removal of venous access) and more use of physical restraint during day shifts compared with night shifts. It is unclear why mechanically ventilated patients who were enrolled in the SLEAP trial received more sedation at night. It is unlikely that the higher nighttime doses reflect more patient distress or agitation, given the similar mean SAS scores and nurse workload during day and night shifts, less use of physical restraint at night, and no increase in adverse events at night. It is possible that nurses or other clinicians perceived that patients, who had been kept awake during the day for physiotherapy, procedures, tests, visits, and weaning, needed additional medications for adequate overnight rest. It is also possible that the expectation of conventional sleep and signs of poor sleep may have prompted nurses to administer more sedative medications at night. An additional possibility is perceived patient discomfort related to a change in ventilation settings at night. Finally, the difference between nighttime and daytime doses may reflect more aggressive weaning of sedatives during the day, because signs of over-sedation were more apparent.

Our finding of increased nocturnal sedation likely reflects general clinical practice, given that SLEAP was a pragmatic trial conducted in 16 centers, and sedation was managed by bedside ICU nurses. Our observation contributes to a very small body of literature on diurnal variation in sedative management and its consequences. Only three studies, all single-center, have evaluated diurnal variation in patient sedation assessment and sedative administration in critically ill adults. In a prospective study evaluating the epidemiology of sedative use in 274 mechanically ventilated adults, Weinert et al. found that daytime nursing staff were more likely to judge patients as “oversedated” compared to their nighttime counterparts, despite only small differences in both sedative dosing and patient behavior [10]. In a study of 140 patients enrolled in the ABC trial [2] Seymour and colleagues observed that benzodiazepine and propofol doses were increased at night on 40 % and 41 % of patient-days, respectively; and an increase in nighttime sedative doses was associated with failed SBTs, coma and delirium [6]. Pisani and colleagues examined dosing patterns of fentanyl, lorazepam and haloperidol in a cohort of 309 patients 60 years and older, and found that doses of lorazepam and haloperidol were higher during the evening shifts (16:00 hours to midnight) than during the day or night shifts [5].

The strengths of our study include protocolized sedation management, multicenter representation, hourly documentation of SAS/RASS and opioid/sedative administration, and self-reported nursing workload assessment. Sedation management in the SLEAP study was pragmatic; the research team did not guide the ICU staff in sedative practices [11]. “Usual care” was assumed, and may vary to a significant degree based on prevailing practice patterns and local culture in different institutions, and the type of ICU (medical vs surgical). These institutional and patient variables may contribute to the increased use of sedatives at night.

Limitations of this study include the observational design of this secondary analysis, and the possibility of omitting important covariates. The generalizability of our findings to shorter-acting sedative agents, such as dexmedetomidine or propofol, may be limited. Our results may not apply to patients experiencing drug withdrawal, which we did not record, or to patients receiving psychotropic medications, used predominantly at night, which have sedative properties of their own. No formal pain scale was used, and sedative/opioid management was guided by patient assessment and SAS or RASS. Finally, the similar SAS scores during the day and night may reflect inaccurate nighttime SAS scoring or reporting by nurses, if they were reluctant to awaken patients. Another possible explanation is insensitivity of the mean SAS score to express small but clinically important differences.

Our findings underscore the need for frequent reassessment of the patient’s sedative and analgesic needs, even during the night. Patients and clinicians would benefit from further research exploring the diurnal variation in sedation, including the reasons for increases in nighttime sedation and barriers to minimizing nighttime sedation.

## Conclusions

Patients enrolled in the SLEAP trial received more opioids and benzodiazepines at night than during the day. The reasons for this remain unclear, and factors such as poor patient sleep, changes in medical personnel, and nighttime changes in ventilator settings may contribute to diurnal sedative variation. Increased nocturnal sedation has important adverse patient consequences, as we found it was independently associated with delayed extubation. Our findings highlight the importance of minimizing sedation at night, as well as during the day.

## Key messages

- In the SLEAP trial, a randomized controlled trial conducted in 16 North American medical-surgical ICUs, mechanically ventilated adults received higher doses of benzodiazepines and opioids at night compared with daytime doses
- Increases in nighttime drug doses were independently associated with failure to meet spontaneous breathing trial (SBT) screening criteria, SBT failure, and the decision not to extubate the patient despite a successful SBT

## Abbreviations

DI, daily interruption; ICU, intensive care unit; OR, odds ratio; PS, protocolized sedation; RASS, Richmond Agitation Sedation Scale; SBT, spontaneous breathing trial; SAS, Sedation Agitation Scale; VAS, visual analogue scale

## Additional file

Additional file 1: Variation in diurnal sedation in mechanically ventilated patients who are managed with a sedation protocol alone or a sedation protocol and daily interruption. (DOC 74 kb)
