# Supplementary material for: Variation in diurnal sedation in mechanically ventilated patients who are managed with a sedation protocol alone or a sedation protocol and daily interruption
Source: Crit Care. 2016 Aug 1;20:233. doi: 10.1186/s13054-016-1405-3 (PMC4968433; doi:10.1186/s13054-016-1405-3)
Supplement: Additional file 1: — Variation in diurnal sedation in mechanically ventilated patients who are managed with a sedation protocol alone or a sedation protocol and daily interruption. (DOC 74 kb) [file 13054_2016_1405_MOESM1_ESM.doc]

Additional File 1.

Variation in diurnal sedation in mechanically ventilated patients who are managed with a sedation protocol alone or a sedation protocol and daily interruption.

Sangeeta Mehta, Maureen Meade, Lisa Burry, Ranjeeta Mallick, Christina Katsios, Dean Fergusson, Peter Dodek, Karen Burns, Margaret Herridge, John W. Devlin, Maged Tanios, Robert Fowler, Michael Jacka, Yoanna Skrobik, Kendiss Olafson, and Deborah Cook, for the SLEAP Investigators and the Canadian Critical Care Trials Group.

Table 1. Richmond Agitation Sedation Score (RASS) and Sedation Agitation Score (SAS) conversion equivalents

| RASS score | SAS Conversion |
| --- | --- |
| +4 | 7 |
| +3 | 6 |
| +2 | 5 |
| +1 | 5 |
| 0 | 4 |
| -1 | 4 |
| -2 | 3 |
| -3 | 3 |
| -4 | 2 |
| -5 | 1 |

Table 2. Night-time and day-time sedative and opioid administration: comparison between the daily interruption and protocolized sedation groups

|  | **Night-time Administration** | |  | **Day-time Administration** | |  |
| --- | --- | --- | --- | --- | --- | --- |
|  | **Protocolized sedation and Interruption**  **N=214** | **Protocolized sedation**  **N=209** | **P value** | **Procolized sedation and Interruption**  **N=214** | **Protocolized sedation**  **N=209** | **P value** |
| **Midazolam equivalents** |  |  |  |  |  |  |
| Total dose/pt (mg)^1^ | 559 (2135) | 534 (2311) | 0.91 | 528 (2164) | 513 (2298) | 0.94 |
| Dose/pt/ shift (mg) | 55 (167) | 43 (152) | 0.01 | 50 (169) | 41 (145) | 0.047 |
| Dose/pt/shift, infusion (mg) | 55 (166) | 43 (152) | 0.01 | 50 (168) | 40.7 (145) | 0.05 |
| Dose/pt/shift, bolus (mg) | 0.52 (3.1) | 0.22 (1.5) | <0.0001 | 0.50 (4.10) | 0.29 (1.89) | 0.03 |
| Number of boluses^2^ | 0.14 (0.72) | 0.08 (0.46) | 0.0008 | 0.12 (0.62) | 0.10 (0.53) | 0.21 |
| Bolus dose (mg) | 0.48 (1.54) | 0.29 (1.25) | 0.17 | 0.49 (1.54) | 0.40 (1.51) | 0.57 |
| **Fentanyl equivalents** |  |  |  |  |  |  |
| Total dose/pt (mcg) ^1^ | 9790 (29790) | 6921 (11564) | 0.19 | 9207 (30201) | 6711 (11774) | 0.26 |
| Dose/pt/shift (mcg) | 965 (2119) | 560 (1065) | <0.0001 | 873 (2180) | 537 (1105) | <0.0001 |
| Dose/pt/shift, infusion (mcg) | 908 (2093) | 521 (1046) | <0.0001 | 810 (2142) | 489 (1061) | <0.0001 |
| Dose/pt/shift, bolus (mcg) | 57 (127) | 40 (85) | <0.0001 | 63 (134) | 48 (119) | <0.0001 |
| Number of boluses^2^ | 1.08 (1.64) | 0.89 (1.52) | <0.0001 | 1.17 (1.72) | 0.94 (1.63) | <0.0001 |
| Bolus dose (mcg) | 58 (83) | 44 (57) | 0.048 | 58 (63) | 46 (56) | 0.04 |

Legend for Table 2. This table shows diurnal variation in benzodiazepine (midazolam equivalents) and opioid (fentanyl equivalents) administration for the two study groups. All data presented as mean (SD).

1 Total dose represents doses received for the duration of the study.

2 Represents total number of boluses received per shift.

Table 3. Night-time and day-time sedative and opioid administration within each group

|  | **Protocolized sedation and Interruption**  **N=214** | |  | **Protocolized sedation**  **N=209** | |  |
| --- | --- | --- | --- | --- | --- | --- |
|  | **Night-time** | **Day-time** | **P value** | **Night-time** | **Day-time** | **P value** |
| **Midazolam equivalents** |  |  |  |  |  |  |
| Total dose/pt (mg) ^1^ | 559 (2135) | 528 (2164) | 0.0005 | 534 (2311) | 513 (2298) | 0.009 |
| Dose/pt/shift (mg) | 55 (167) | 50 (169) | 0.0005 | 43 (152) | 41 (145) | 0.10 |
| Dose/pt/shift, infusion (mg) | 55 (166) | 50 (168) | 0.0006 | 43 (152) | 41 (145) | 0.09 |
| Dose/pt/shift, bolus (mg) | 0.52 (3.1) | 0.50 (4.10) | 0.95 | 0.22 (1.5) | 0.29 (1.89) | 0.06 |
| Number of boluses ^2^ | 0.14 (0.72) | 0.12 (0.62) | 0.17 | 0.08 (0.46) | 0.10 (0.53) | 0.18 |
| Bolus dose (mg) | 0.48 (1.54) | 0.49 (1.54) | 0.94 | 0.29 (1.25) | 0.40 (1.51) | 0.26 |
| **Fentanyl equivalents** |  |  |  |  |  |  |
| Total dose/pt (mcg) ^1^ | 9790 (29790) | 9207 (30201) | 0.003 | 6921 (11564) | 6711 (11774) | 0.33 |
| Dose/pt/shift (mcg) | 965 (2119) | 873 (2180) | 0.0007 | 560 (1065) | 537 (1105) | 0.16 |
| Dose/pt/shift, infusion (mcg) | 908 (2093) | 810 (2142) | 0.0002 | 521 (1046) | 489 (1061) | 0.02 |
| Dose/pt/shift, bolus (mcg) | 57 (127) | 63 (134) | 0.02 | 40 (85) | 48 (119) | <0.0001 |
| Number of boluses ^2^ | 1.08 (1.64) | 1.17 (1.72) | 0.001 | 0.89 (1.52) | 0.94 (1.63) | 0.06 |
| Bolus dose (mcg) | 58 (83) | 58 (63) | 0.98 | 44 (57) | 46 (56) | 0.60 |

Legend for Table 3. This table shows diurnal variation in benzodiazepine (midazolam equivalents) and opioid (fentanyl equivalents) administration within each study group. Data presented as mean (SD).

1 Total dose represents doses received for the duration of the study.

2 Represents total number of boluses received per shift
